# Influência do Bem-estar Espiritual na Pressão Arterial, Hemodinâmica Central e Função Endotelial

**DOI:** 10.36660/abc.20210886

**Published:** 2022-10-05

**Authors:** Maria Emília Figueiredo Teixeira, Priscila Valverde de Oliveira Vitorino, Andrea A. Brandão, Ana Luiza Lima Souza, Talles Marcelo Gonçalves de Andrade Barbosa, Roberto Esporcatte, Mário Henrique Elesbão de Borba, Álvaro Avezum, Weimar Kunz Sebba Barroso

**Affiliations:** 1 Universidade Federal de Goiás Liga de Hipertensão Arterial Goiânia GO Brasil Universidade Federal de Goiás – Liga de Hipertensão Arterial , Goiânia , GO – Brasil; 2 Universidade Federal de Goiás Programa de Pós-Graduação em Ciências da Saúde Goiânia GO Brasil Universidade Federal de Goiás – Programa de Pós-Graduação em Ciências da Saúde , Goiânia , GO – Brasil; 3 Pontifícia Universidade Católica de Goiás Escola de Ciências Sociais e da Saúde Goiânia GO Brasil Pontifícia Universidade Católica de Goiás – Escola de Ciências Sociais e da Saúde , Goiânia , GO – Brasil; 4 Universidade do Estado do Rio de Janeiro Departamento de Doenças do Tórax Rio de Janeiro RJ Brasil Universidade do Estado do Rio de Janeiro – Departamento de Doenças do Tórax , Rio de Janeiro , RJ – Brasil; 5 Pontifícia Universidade Católica de Goiás Escola de Ciências Exatas e da Computação Goiânia GO Brasil Pontifícia Universidade Católica de Goiás – Escola de Ciências Exatas e da Computação (ECEC), Goiânia , GO – Brasil; 6 Cardio Clínica do Vale Lajeado RS Brasil Cardio Clínica do Vale , Lajeado , RS – Brasil; 7 International Research Center Hospital Alemão Oswaldo Cruz São Paulo SP Brasil International Research Center , Hospital Alemão Oswaldo Cruz , São Paulo , SP – Brasil

**Keywords:** Espiritualidade, Religião e Medicina, Pressão Arterial, Hemodinâmica, Endotélio/fisiologia, Valores Sociais, Valor da Vida, Qualidade de Vida

“ *Science is not only compatible with spirituality; it is a profound source of spirituality.* ”

**Carl Sagan**

## Introdução

Espiritualidade ^1^ e religiosidade (E/R) são aspectos culturais presentes desde os primórdios da existência humana. Vistas por muito tempo como opostas à ciência, apenas recentemente, E/R ganharam relevância no âmbito da saúde. ^1^

As definições de E/R estão em evolução constante, de acordo com as necessidades de adequação a novos conhecimentos. Religião tem sido relacionada, contemporaneamente, com aspectos organizacionais, institucionais e dogmáticos, ou seja, o contato com a deidade acontece por meio de formatos pré-determinados e específicos para cada segmento religioso. ^2^ Espiritualidade, termo mais amplo, engloba a busca pelo bem-estar pessoal, psicológico, espiritual e nas relações pessoais. Para o Departamento de Estudos em Espiritualidade e Medicina Cardiovascular (DEMCA) da Sociedade Brasileira de Cardiologia (SBC), “espiritualidade é um conjunto de valores morais, mentais e emocionais que norteiam pensamentos, comportamentos e atitudes nas circunstâncias da vida de relacionamento intra e interpessoal”. ^3^

A hipertensão arterial (HA) é doença de elevada prevalência e principal fator de risco para outras doenças cardiovasculares, ^4^ o que a torna a principal causa direta e indireta de mortalidade em todo o planeta. ^5^ Por ser uma doença multifatorial, seu tratamento contempla medidas farmacológicas e não farmacológicas, ^6^ que incluem medidas voltadas para o bem-estar físico e mental. ^7 , 8^

Práticas que busquem o bem-estar espiritual, aliadas ou não à religiosidade, tem sido relacionadas ao bom controle de muitas doenças, ^9^ além de redução de mortalidade em diversas situações. ^10^ Existem evidências da associação entre E/R e desfechos positivos em cardiologia, tais como Doença Arterial Coronariana (DAC), ^11^ Insuficiência Cardíaca (IC) ^12^ e HA. ^13 , 14^

Estudos sobre E/R são ainda incipientes, e a maioria observacionais. Em conjunto, E/R foram associadas a melhores hábitos de vida (menos sedentarismo, etilismo ou tabagismo), ^15^ menores valores pressóricos, menor risco de HA ^16^ e melhor adesão terapêutica. ^17^ Bem-estar espiritual isoladamente pode ser um fator cardioprotetor, por estar relacionado a menores níveis de pressão arterial (PA), glicemia, triglicérides e LDL colesterol. ^18^ Por outro lado, um estudo observou maior probabilidade de HA associada à maior frequência de orações, mas menor probabilidade de HA associada a variáveis de propósito e perdão. ^19^

Diante disso, torna-se relevante avaliar o efeito de uma intervenção voltada para o bem-estar espiritual no controle pressórico e de outros parâmetros hemodinâmicos. Este artigo descreve o método de um ensaio clínico para avaliar uma intervenção em espiritualidade sobre a PA periférica e central (PAC), parâmetros de rigidez arterial e função endotelial, em hipertensos estágios 1 e 2 de baixo ou moderado risco cardiovascular antes e após 12 semanas de seguimento dentro de cada grupo (controle [GC] e intervenção [GI]) e entre os grupos.

## Métodos

### Tipo e local do estudo

Ensaio clínico randomizado de não inferioridade cuja coleta de dados será realizada na Liga de Hipertensão Arterial da Universidade Federal de Goiás (UFG) e na Universidade do Estado do Rio de Janeiro. O protocolo será devidamente cadastrado no Registro Brasileiro de Ensaios Clínicos (ReBEC).

### População, amostra e amostragem

A população do estudo será composta por adultos hipertensos (estágio 1 ou 2) com risco cardiovascular baixo e moderado, em uso estável de medicação anti-hipertensiva há mais de trinta dias, avaliada por medidas pressóricas da última consulta.

A amostra foi calculada utilizando o OpenEpi. Foi considerada uma pressão arterial sistólica (PAS) no GI de 130,9 ± 9,2 e no GC de 135,81 ± 9,3 mmHg, ^20^ com intervalo de confiança de 95% e poder de teste de 80% com 54 participantes em cada grupo.

Serão excluídos hipertensos estágio 3 [PAS ≥ 180 mmHg e/ou PA Diastólica (PAD) ≥ 110 mmHg].

Após a inclusão, serão retirados aqueles que se recusarem a realizar qualquer procedimento e/ou apresentarem elevação pressórica durante o seguimento que impossibilite manter a conduta proposta sem alteração da medicação.

### Recrutamento e randomização dos pacientes do estudo

As equipes envolvidas se reunirão para apresentação, discussão do projeto e treinamento para execução rigorosa do protocolo e uniformização na abordagem aos pacientes, com vistas a garantir rigor metodológico.

Os pacientes serão selecionados pelo último registro da PA em prontuário, verificando o estágio de HA e o risco cardiovascular em que se encontram e serão convidados a participarem no estudo por meio de ligação telefônica. Aqueles que aceitarem participar, serão chamados para a visita inicial.

### Visita inicial (V0)

Os participantes serão randomizados no site *www.randomizer.* org para um dos dois grupos.

Todos receberão orientações relacionadas a hábitos de vida saudáveis e serão submetidos à anamnese, exame clínico e entrevista para preenchimento de quatro questionários: Durel, ^21 , 22^ predisposição ao perdão, ^23^ escala de gratidão ^24^ e escala de bem-estar espiritual. ^25^ Todo processo será feito por pesquisadores previamente treinados, que utilizarão um roteiro padronizado, de forma a uniformizar a consulta e as orientações.

Além disso, será aferida PA casual por método oscilométrico com aparelho Dyna- MAPA AOP (Cardios, Brasil), que fornece valores de PA periférica e central (PAC), velocidade de onda de pulso (VOP) obtida por algoritmo e equação matemática ARC SOLVER e expressa em metros/segundo, e Augmentation Index corrigido para 75% da frequência cardíaca (AIx). ^26 , 27^ PA periférica será obtida conforme recomendação da Diretriz Brasileira de HA 2020. ^8^

A Monitorização Ambulatorial da Pressão Arterial (MAPA) será executada com aparelho Dyna-MAPA (Cardios, Brasil), seguindo as recomendações da mais recente Diretriz Brasileira de MAPA. ^28^ A dilatação fluxo-mediada (DFM) será realizada em aparelho de ultrassom de alta resolução automatizado e com braço robótico para a precisa localização e medida da artéria braquial (UNEX EF 38G), de acordo com a técnica de Celermajer et al., ^29^ preconizada pela International Brachial Artery Reactivity *Task* Force. ^30 , 31^ A DFM é o atual padrão ouro para avaliar função endotelial; o endotélio saudável apresenta FMD > 10%, e valores abaixo a esse são preditivos de maior risco cardiovascular. ^32^

### Visita intermediária (V1)

A V1 ocorrerá seis semanas após V0 por ligação telefônica para todos, com o intuito de avaliar o bem-estar individual e estimular o seguimento ativo na realização da intervenção para aqueles do GI, esclarecendo a importância da execução diária da atividade proposta e o questionamento sobre possíveis fatores que possam limitar o acesso ou a compreensão. Pacientes com níveis pressóricos acima de 180/110 mmHg ou com sintomas como dor precordial ou cefaleia intensa terão consultas presenciais agendadas.

### Visita final (V2)

A V2 será feita para ambos os grupos ao final do treinamento proposto, com janela aceitável de ± três dias. Todos serão submetidos aos mesmos procedimentos de V0.

### Grupo intervenção

A intervenção terá início na manhã seguinte à finalização de V0, com duração de 12 semanas. Esse tempo de seguimento foi utilizado em estudos com intervenções não medicamentosas em hipertensos, e demonstrou ser suficiente para obter mudanças nos valores pressóricos. ^33 , 34^ A intervenção consistirá em uma sequência de vídeos previamente gravados, mensagens, tarefas curtas relacionadas ao tema do vídeo e dias de folga ( Tabela 1 ). Serão abordados assuntos ligados à espiritualidade, como perdão, gratidão, otimismo, propósito de vida e bem-estar espiritual. O conteúdo será disponibilizado diariamente em um aplicativo para smartphone, que registrará as atividades realizadas por cada participante.

**Tabela 1:** Sequência de tarefas por dia da semana do grupo intervenção

Dia	Tarefa	Dia	Tarefa	Dia	Tarefa	Dia	Tarefa
1	V 1	22	AT 6	43	F	64	MR 22
2	MR 1	23	MR 8	44	V 9	65	AT 21
3	AT 1	24	F	45	MR 15	66	F
4	F	25	V 6	46	AT 14	67	V 12
5	V 2	26	MR 9	47	MR 16	68	MR 23
6	MR 2	27	AT 7	48	AT 15	69	AT 22
7	AT 2	28	MR 10	49	MR 17	70	MR 24
8	F	29	AT 8	50	AT 16	71	AT 23
9	V 3	30	F	51	F	72	F
10	MR 3	31	V 7	52	V 10	73	V 13
11	AT 3	32	MR 11	53	MR 18	74	MR 25
12	F	33	AT 9	54	AT 17	75	AT 24
13	V 4	34	AT 10	55	MR 19	76	MR 26
14	MR 4	35	MR 12	56	F	77	F
15	AT 4	36	AT 11	57	V 11	78	V 14
16	MR 5	37	AT 12	58	MR 20	79	MR 27
17	F	38	F	59	AT 18	80	MR 28
18	V 5	39	V 8	60	MR 21	81	F
19	MR 6	40	MR 13	61	AT 19	82	V 15
20	AT 5	41	AT 13	62	F	83	MR 29
21	MR 7	42	MR 14	63	AT 20	84	AT 25

*V: vídeo; MR: mensagem de reflexão; AT: atividade; F: folga.*

Serão considerados participantes com adesão satisfatória à intervenção aqueles que realizarem pelo menos 75% das tarefas propostas.

### Grupo controle

O GC será acompanhado nos serviços dentro da mesma periodicidade definida para o GI. Se observamos resultados superiores no GI, o GC receberá o mesmo tratamento após a conclusão do estudo.

### Análise dos dados

Os dados coletados serão analisados com a utilização do Software Stata 14.0. As variáveis qualitativas serão apresentadas com média e desvio padrão e as quantitativas com média e desvio padrão ou mediana e intervalo interquartil.

A distribuição dos dados das variáveis será verificada quanto à normalidade com o teste de Kolmogorov Smirnov. Serão aplicados testes de acordo com a normalidade dos dados para a avaliação entre os grupos (intervenção e controle) e intra-grupo (antes e depois) para cada um deles. Se o caso, será realizada a análise “intention-to-treat”, e somente com pacientes que completaram o protocolo do estudo.

O desfecho primário do estudo será a pressão arterial sistólica periférica e os desfechos secundários serão: a PASc, a VOP, a PAS média e a DFM.

### Aspectos éticos

O projeto foi submetido e aprovado pelo Comitê de Ética em Pesquisa do Hospital das Clínicas da UFG.

## Conclusão

Esta pesquisa buscará compreender o efeito de uma intervenção do estímulo e treinamento para a busca do bem-estar espiritual por meio da propensão ao perdão, otimismo, gratidão e propósito de vida no comportamento da pressão arterial.

O estudo poderá impactar positivamente a prática clínica ao trazer embasamento para uma abordagem não farmacológica para o tratamento da HA além de ser, em nosso conhecimento, um dos primeiros ensaios clínicos com esse desenho.
