# Supplementary material for: Influência do Bem-estar Espiritual na Pressão Arterial, Hemodinâmica Central e Função Endotelial
Source: Arq Bras Cardiol. 2022 Oct 5;119(4):622–6. [Article in Portuguese] doi: 10.36660/abc.20210886 (PMC9563879; doi:10.36660/abc.20210886)
Supplement: Translation

# Translation: Influence of Spiritual Well-Being on Blood Pressure, Central Hemodynamics and Endothelial Function

**Authors:** Maria Emília Figueiredo Teixeira, Priscila Valverde de Oliveira Vitorino, Andrea A. Brandão, Ana Luiza Lima Souza, Talles Marcelo Gonçalves de Andrade Barbosa, Roberto Esporcatte, Mário Henrique Elesbão de Borba, Álvaro Avezum, Weimar Kunz Sebba Barroso

**Affiliations:** 1 Brazil Universidade Federal de Goiás – Liga de Hipertensão Arterial, Goiânia, GO – Brazil; 2 Brazil Universidade Federal de Goiás – Programa de Pós-Graduação em Ciências da Saúde, Goiânia, GO – Brazil; 3 Brazil Pontifícia Universidade Católica de Goiás – Escola de Ciências Sociais e da Saúde, Goiânia, GO – Brazil; 4 Brazil Universidade do Estado do Rio de Janeiro – Departamento de Doenças do Tórax, Rio de Janeiro, RJ – Brazil; 5 Brazil Pontifícia Universidade Católica de Goiás – Escola de Ciências Exatas e da Computação (ECEC), Goiânia, GO – Brazil; 6 Brazil Cardio Clínica do Vale, Lajeado, RS – Brazil; 7 Brazil International Research Center, Hospital Alemão Oswaldo Cruz, São Paulo, SP – Brazil

**Keywords:** Spirituality, Religion and Medicine, Blood Pressure, Hemodynamic, Endothelium/physiology, Social Values, Value of Life, Quality of Life

“ *Science is not only compatible with spirituality; it is a profound source of spirituality.* ”

**Carl Sagan**

## Introduction

Spirituality^1^ and religiosity (S/R) are cultural aspects that have existed since the beginnings of human existence. Having been long considered as opponent to science, only recently, S/R have gained importance in the context of health.^1^

Definitions of S/R have been constantly evolving, according to the need for adaptation to new knowledge. Today, religion has been related to organizational, institutional and dogmatic aspects, that is, the contact with deity occurs through predetermined formats, specific to each religious segment.^2^ Spirituality is a wider term, encompassing a quest for personal, psychological and spiritual well-being, and good-quality interpersonal relationships. According to the Department of Studies in Spirituality and Cardiovascular Medicine (DEMCA, *Departamento de Estudos em Espiritualidade e Medicina Cardiovascular* ) of the Brazilian Cardiology Society, “spirituality is a set of moral, mental and emotional values that guide thoughts, behaviors and attitudes in life circumstances of intra and interpersonal relationships”.^3^

Arterial hypertension (AH) is a highly prevalent disease and the main risk factor for cardiovascular diseases,^4^ and hence the main direct and indirect cause of mortality in the world.^5^ Since AH is a multifactorial disease, its treatment encompasses both pharmacological and non-pharmacological strategies,^6^ focusing on physical and mental well-being.^7 , 8^

Practices that promote spiritual well-being, allied or not to religiosity, have been associated with the good control of many diseases^9^ and reduction of mortality in several situations.^10^ There has been evidence of an association between E/R and positive outcomes in heart diseases, such as coronary artery disease (CAD),^11^ heart failure (HF),^12^ and AH.^13 , 14^

S/R studies are still incipient, and mostly observational. In general, E/R have been associated with better life habits (less sedentarism, alcohol consumption and smoking),^15^ lower blood pressure (BP), lower risk for AH,^16^ and better treatment compliance.^17^ Spiritual well-being alone may be a cardioprotective factor, as it is correlated with lower levels of BP, fasting glucose, triglycerides and low-density lipoprotein (LDL) cholesterol.^18^ On the other hand, an observational study reported an increased likelihood of AH associated with higher frequency of prayer, but a lower likelihood of hypertension associated with variables for meaning and forgiveness.^19^

In light of this, it is important to evaluate the effect of an intervention focusing on spiritual well-being on the control of BP and other hemodynamic parameters. This paper describes the methodology of a clinical trial to evaluate the effects of an intervention in spirituality on peripheral and central BP (cBP) (parameters of arterial stiffness and endothelial function) in hypertensive patients in stages 1 and 2, at low or moderate cardiovascular risk. The parameters of a control group (CG) and an intervention group (IG) will be analyzed before and after 12 months of follow-up in each group and between the groups.

## Methods

### Type and place of study

This is a randomized non-inferiority trial; data collection will be carried out in the University of Goias Hypertension League ( *Liga de Hipertensão Arterial da Universidade Federal de Goiás* ) and in the Rio de Janeiro State University ( *Universidade do Estado do Rio de Janeiro* ). The protocol will be registered on the Brazilian Registry of Clinical Trials (ReCEB, *Registro Brasileiro de Ensaios Clínicos* ).

### Population, sample, and sampling

The study population will be composed of hypertensive adults (stage 1 or 2) at low or moderate cardiovascular risk, under stable treatment with antihypertensive medications for more than 30 days, which was evaluated by BP measures taken during the last visit.

Sample calculation was performed using the OpenEpi calculator. A systolic blood pressure (SBP) of 130.9 ± 9.2 and 135.81 ± 9.3 mmHg^20^ was considered in the IG and the CG, respectively, with a 95% confidence interval and an 80% power of test, with 54 participants in each group.

Stage 3 hypertensive patients (SBP ≥ 180 mmHg and/or diastolic blood pressure [DBP] ≥ 110mmHg) will be excluded.

After patient enrollment, patients who decline to participate in any procedure, and/or show a rise in BP during the follow-up, preventing their participation in the study without changing the drug regimen will be excluded.

### Patient recruitment and randomization

The staffs involved in the study will meet for the presentation and discussion about the project, and training of the members for the correct execution of the protocol and uniformization of patient approach, for the sake of methodological rigor.

Patients will be selected based on the last BP recorded in the medical record, and on the classification of AH stage and cardiovascular risk, and will be invited to participate in the study by telephone. Those who accept to participate will be invited for the first visit.

### First visit (V0)

Randomization of participants into one of the two groups will be performed by using www. randomizer.org.

All patients will receive information about healthy life habits. Then, the medical history will be taken, and clinical examination and interview will be performed for completion of four questionnaires – Durel,^21 , 22^ willingness to forgive,^23^ the gratitude questionnaire,^24^ and the spiritual well-being questionnaire.^25^ All procedures will be performed by trained investigators, following the same script, to standardize the visits and instructions.

In addition, casual BP will be measured by oscillometry using a Dyna-MAPA AOP device (Cardios, Brazil), that provides measurements of peripheral BP, central BP (cBP), pulse wave velocity (PWV) (using an algorithm and ARC SOLVER equation and expressed as meter/second), and the augmentation index adjusted to 75% of the heart rate (AIx).^26 , 27^ Peripheral BP measurements will be obtained according to the latest Brazilian guidelines on hypertension.^8^

Ambulatory blood pressure monitoring (ABPM) will be performed using a Dyna-MAPA AOP device (Cardios, Brazil), following the latest Brazilian guidelines on ABPM.^28^

Flow-mediated dilation (FMD) will be determined by a high-resolution ultrasound scanner and a robotic arm to obtain a precise positioning and measurement of the brachial artery (UNEX EF 38G), according to the technique proposed by Celermajer et al.^29^ and recommendations of the International Brachial Artery Reactivity Task Force.^30 , 31^ FMD is the current gold standard method to evaluate endothelial function; a FMD > 10% indicates a healthy endothelium, and values below that are predictive of increased cardiovascular risk.^32^

### Intermediate visit (V1)

An intermediate visit will be held six weeks after V0, by telephone call, to verify patient well-being and encourage adherence to the intervention (IG), highlighting the importance of performing the daily activities proposed and clarifying possible doubts. In-person visits will be scheduled with patients with BP levels above 180/110mmHg and patients with symptoms such as precordial pain or severe headache.

### Final visit (V2)

The final visit will be held for both IG and CG after the program proposed, with an acceptable time window of ± three days. All patients will undergo the same procedures of V0.

### Intervention group

The intervention will begin in the morning after the V0 and have a duration of 12 weeks. This follow-up period was used in previous non-pharmacological intervention studies with hypertensive patients and shown to be sufficient to detect changes in BP.^33 , 34^

The intervention will consist of a series of previously recorded videos, messages, short tasks related to the subject of the video and days off ( Table 1 ). Themes related to spirituality, forgiveness, gratitude, optimism, life purpose, and spiritual well-being will be addressed. The content will be available through a smartphone app, which will register the activities performed by each participant daily.

**Table 1:** Sequence of tasks by weekday for the intervention group

Day	Task	Day	Task	Day	Task	Day	Task
1	V 1	22	AT 6	43	R	64	TM 22
2	TM 1	23	TM 8	44	V 9	65	AT 21
3	AT 1	24	R	45	TM 15	66	R
4	R	25	V 6	46	AT 14	67	V 12
5	V 2	26	TM 9	47	TM 16	68	TM 23
6	TM 2	27	AT 7	48	AT 15	69	AT 22
7	AT 2	28	TM 10	49	TM 17	70	TM 24
8	R	29	AT 8	50	AT 16	71	AT 23
9	V 3	30	R	51	R	72	R
10	TM 3	31	V 7	52	V 10	73	V 13
11	AT 3	32	TM 11	53	TM 18	74	TM 25
12	R	33	AT 9	54	AT 17	75	AT 24
13	V 4	34	AT 10	55	TM 19	76	TM 26
14	TM 4	35	TM 12	56	R	77	R
15	AT 4	36	AT 11	57	V 11	78	V 14
16	TM 5	37	AT 12	58	TM 20	79	TM 27
17	R	38	R	59	AT 18	80	TM 28
18	V 5	39	V 8	60	TM 21	81	R
19	TM 6	40	TM 13	61	AT 19	82	V 15
20	AT 5	41	AT 13	62	R	83	TM 29
21	TM 7	42	TM 14	63	AT 20	84	AT 25

*V: video; TM: thinking message; AT: activity; R: respite*

The adherence to the intervention will be considered satisfactory when 75% or more of the proposed tasks are completed.

### Control group

The CG will be monitored at the same frequency as the IG. If the results in the IG are significantly better than in the CG, the latter will receive the same treatment at the end of the study period.

### Statistical analysis

Data will be analyzed using the Software Stata 14.0. Qualitative variables will be expressed as mean and standard deviation, and quantitative variables as mean and standard deviation or median and interquartile range.

Normality of data distribution will be tested by the Kolmogorov-Smirnov test. Statistical tests will be applied according to normality of the data for between-group (IG and CG) and within-group comparisons (before and after). If this is the case, the intention-to-treat analysis will be performed, and only with patients who complete the study protocol.

The primary outcome will be peripheral systolic blood pressure and the secondary outcomes will be: cSBP, PWV, mean SBP and FMD.

### Ethical aspects

The project was submitted and approved by the Ethics Committee of the UFG General Hospital.

## Conclusion

This research will investigate the effects of an intervention based on encouragement and training to achieve spiritual well-being through propensity to forgive, optimism, gratitude, and life purpose on BP behavior.

The study will have a positive impact on clinical practice by presenting the basis for a non-pharmacological approach to the treatment of AH. Also, to our knowledge, this is one of the first clinical trials with this design.
